# Neonatal McCune–Albright syndrome with systemic involvement: a case report

**DOI:** 10.1186/s13256-015-0689-2

**Published:** 2015-09-04

**Authors:** Rita Lourenço, Patrícia Dias, Raquel Gouveia, Ana Berta Sousa, Graça Oliveira

**Affiliations:** 1 Neonatal Intensive-Care Unit, Department of Pediatrics, Hospital de Santa Maria, Centro Hospitalar Lisboa Norte, Centro Académico de Medicina de Lisboa, Lisbon, Portugal; 2 Genetics Service, Department of Pediatrics, Hospital de Santa Maria, Centro Hospitalar Lisboa Norte, Centro Académico de Medicina de Lisboa, Lisbon, Portugal

**Keywords:** Cushing’s syndrome, *GNAS* gene mutations, McCune–Albright syndrome, Metyrapone

## Abstract

**Introduction:**

McCune–Albright syndrome is a rare sporadic disease characterized by fibrous bone dysplasia, café-au-lait skin spots and variable hyperfunctional endocrinopathies. McCune–Albright syndrome is caused by somatic postzygotic activating mutations in the *GNAS* gene that produce a broad spectrum of effects.

**Case presentation:**

We report a case of McCune–Albright syndrome with multi-organ manifestations in the neonatal period. A newborn preterm black girl was referred to our Neonatal Intensive Care Unit at the age of 17 days for suspected extrahepatic cholestasis. On clinical examination she presented failure to thrive, jaundice, hypertension, marked hypotonia and café-au-lait spots on her back and lower limbs. An abdominal ultrasound excluded extrahepatic causes of cholestasis but revealed bilateral serpiginous adrenal hyperplasia. These clinical findings suggested a diagnosis of McCune–Albright syndrome with multi-organ involvement. Laboratory data confirmed adrenocorticotropic hormone-independent Cushing’s syndrome, hyperthyroidism, cholestasis and elevated transaminases. Ventricular hypertrophy was demonstrated by echocardiography. The baby girl underwent medical treatment of Cushing’s syndrome with metyrapone which was followed by a rapid recovery. A mosaic activating *GNAS* gene mutation was found on DNA extracted from a buccal swab sample. However, she died at 4 months due to a respiratory infection.

**Conclusion:**

In the neonatal period the diagnosis of McCune–Albright syndrome depends on having a high index of suspicion and café-au-lait spots may be the clue for the diagnosis.

## Introduction

McCune–Albright syndrome (MAS) is a rare sporadic disease characterized by bone fibrous dysplasia, café-au-lait (CAL) skin spots and a variable association of hyperfunctional endocrinopathies [1]. The estimated prevalence ranges between 1/100,000 and 1/1,000,000 [2].

MAS manifestations are due to somatic activating mutations in the *GNAS* gene which codes for the alpha subunit of the stimulatory G-protein (protein Gsα) that is involved in intracellular cyclic adenosine monophosphate (cAMP) production [3]. A wide spectrum of extraskeletal manifestations can be found in MAS as would be expected from the broad tissue distribution of Gsα and the mosaic distribution of the mutation [4].

Cushing’s syndrome may develop at any time in childhood but affects a minority of patients with MAS and has a quite heterogeneous natural history, ranging from spontaneous resolution to the need for adrenalectomy or even death [5].

Liver and cardiac diseases are additional less common extraskeletal manifestations of MAS and are markers of prognosis in children with Cushing’s syndrome, which may suggest the need for prompt adrenalectomy [6].

Severe neonatal presentation of MAS is rare. We report a case of MAS in the neonatal period with multi-organ manifestations.

## Case presentation

A 17-day-old black female neonate was referred to our Neonatal Intensive Care Unit (NICU) for suspected biliary atresia on the basis of cholestasis with acholia.

She was the first child of healthy non-consanguineous parents. Prenatal care was minimal. She was born at 35 weeks and 5 days, weighing 1590g (below third centile). Apgar scores were 8 and 9 at 1 and 5 minutes respectively. Jaundice was noted from the second day onwards and acholic stools were occasionally observed. Abdominal ultrasonography prompted concern for a choledochal cyst and apparent atresia. Hepatobiliary scintigraphy was performed but the results were inconclusive.

She also had a history of respiratory distress requiring oxygen therapy as well as hypertension treated with propranolol since day 14, at which an echocardiogram was reported as normal.

**Fig. 1:**
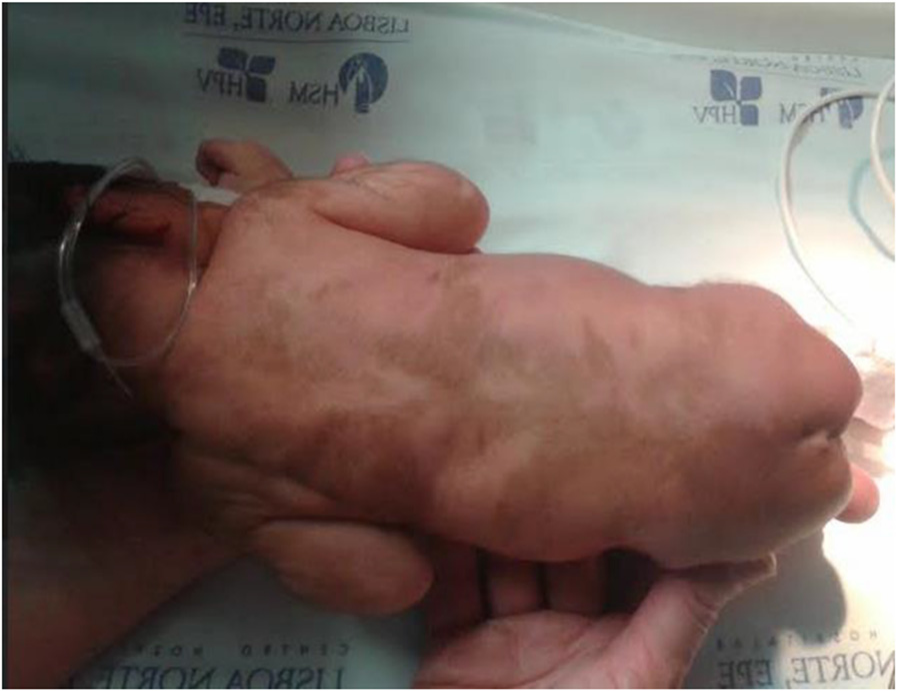
Café-au-lait spots extending from the midline to lateral left and right dorsum, occupying most of its extension

On clinical examination she was small for gestational age (length 39.5cm, weight 1545g, head circumference 28.5cm, all below the third centile). Her blood pressure was elevated (118/90mmHg, above the 95th centile). She had marked hypotonia, well-defined CAL spots following Blaschko’s lines on her back and lower limbs, heart murmur on cardiac auscultation and palpable liver edge 2.5cm below the costal margin (Fig. 1).

### Outcome and investigations

Laboratory findings confirmed cholestasis and abnormal liver function: total bilirubin 10.8mg/dL (normal <1mg/dL); conjugated bilirubin 9.5mg/dL (normal <0.3mg/dL); aspartate aminotransferase (AST) 211U/L (normal <34U/L); alanine aminotransferase (ALT) 623U/L (normal 12 to 78U/L); gamma-glutamyltransferase (γGT) 1219UI/L (normal <38UI/L); total cholesterol 343mg/dL (normal <190mg/dL); triglycerides 240mg/dL (normal <150mg/dL); total serum proteins, albumin, activated partial thromboplastin time, prothrombin time and fibrinogen were normal. Further investigations ruled out the most common causes of cholestasis (metabolic disease included in the neonatal screening, α1-antitrypsin deficiency, and cystic fibrosis). Because of ongoing cholestasis, ultrasonography of her abdomen was repeated; it demonstrated the absence of a choledochal cyst and normal biliary tree but revealed bilateral serpiginous adrenal hyperplasia.

The association of abnormal skin pigmentation, cholestasis and adrenal hyperplasia led to genetic assessment and diagnosis of MAS.

A chest radiograph showed cardiomegaly. Repeated echocardiograms were performed revealing left ventricular hypertrophy, with initial thickness of the interventricular septum of 5.2mm (Z-Score 2.06) reaching a maximum of 7mm (Z-Score 3.44). Systemic hypertension (systolic pressures 90 to 145mmHg, diastolic pressures 55 to 105mmHg) was confirmed. Primary renal disease was excluded by normal values of plasma renin activity and aldosterone, as well as renal imaging studies; her urinary catecholamines were normal. Echocardiography also documented the presence of pulmonary hypertension (systolic pulmonary artery pressure 55 to 60mmHg) at 73 days of age, with progressive improvement until normalization.

As the baby presented markedly decreased muscle tone, growth restriction, hypertension and hyperglycemia (150 to 345mg/dL), other endocrine investigations were performed. Data showed hyperthyroidism: thyroid-stimulating hormone (TSH) 0.01uU/mL (normal 0.5 to 6.0uU/mL); free thyroxine (T_4_) 2.51ng/dL (normal 0.8 to 1.76ng/dL); free triiodothyronine (T_3_) 3pg/dL (normal 2.3 to 4.2pg/dL). Levels of antithyroid antibodies were undetectable. Urinary free cortisol was increased (>75.0ug/dL/24 hours) and she had high serum cortisol without circadian variation (73.7ug/dL at 8 a.m., normal 4.3 to 23; 93.3ug/dL at 11 p.m., normal 2.4 to 13.6ug/dL) and no suppression on high-dose dexamethasone (108.4ug/dL). Her adrenocorticotropic hormone (ACTH) level was normal (9.2pg/mL, normal 0 to 46pg/mL). These results were concordant with the diagnosis of ACTH-independent Cushing’s syndrome.

**Fig. 2:**
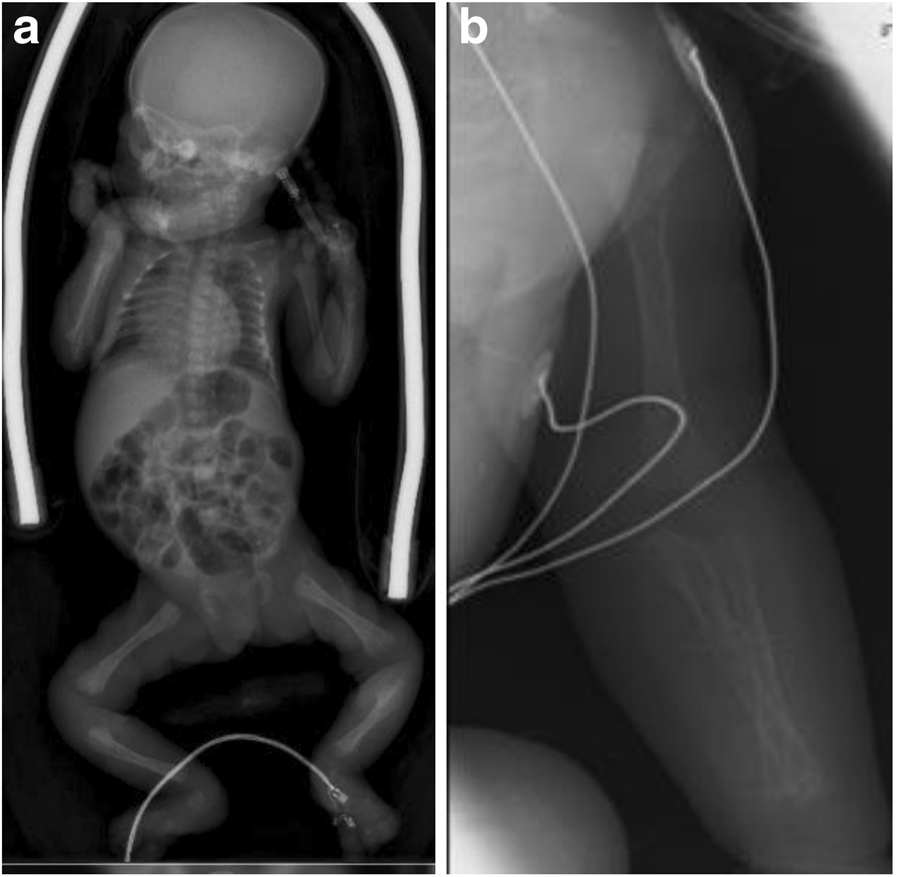
**a** Radiographic appearance of bone fibrodysplasia. An important cardiomegaly is also observed. **b** Diaphyseal fracture of the ulna. In McCune–Albright syndrome fractures occur as a result of osteopenia associated with cortical thinning

A skeletal survey revealed generalized reduction of bone density, irregular ossification with areas of radiolucence surrounded by sclerosis most evident in her radius and ulna (Fig. 2).

Molecular diagnosis was possible in DNA extracted from a buccal swab sample, followed by amplification of the *GNAS* gene and direct sequencing. The activating mutation c.602G> A, leading to p. Arg201 to His, was identified in mosaic, confirming the clinical diagnosis of MAS.

### Treatment and follow-up

**Fig. 3:**
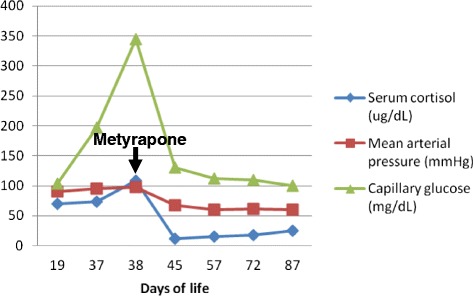
Metyrapone effectiveness in the control of serum cortisol

The baby girl underwent medical treatment of Cushing’s syndrome with metyrapone (six daily doses of 40mg) from day 38 of age which was followed by rapid normalization of cortisol levels with glycemic and mean arterial pressure improvement (Fig. 3). Her growth velocity accelerated, producing gradual catch-up growth.

Her thyroid function was controlled with methimazole 1mg/kg three times a day.

Otherwise, her liver function test levels remained high (AST 709U/L, normal <34U/L; ALT 1656U/L, normal 12 to 78U/L; γGT 1541UI/L, normal <38UI/L; total bilirubin 7.65mg/dL, normal <1mg/dL; conjugated bilirubin 6.77mg/dL, normal <0.3mg/dL; total cholesterol 534mg/dL, normal <190mg/dL; triglycerides 198U/L mg/dL, normal <150mg/dL). A trial of phenobarbital and ursodeoxycholic acid was initiated in an effort to stimulate bile flow, but jaundice persisted.

Hyperglycemia required insulin infusion from day 37 to day 47 of life and normalized after Cushing’s syndrome control.

Hypertension control required the combination of two antihypertensive agents (propranolol and captopril) but normalized only after the introduction of metyrapone. Her echocardiogram showed a decrease in ventricular hypertrophy.

She had developmental delay with no apparent vision or hearing impairment. Magnetic resonance imaging showed hypoplasia of the cerebellar vermis.

The baby girl remained hospitalized due to oxygen dependence. At 4 months she was transferred to the Pediatric Intensive Care Unit for acute respiratory failure in the context of a pulmonary infection leading to her death. No infectious agent was isolated.

**Fig. 4:**
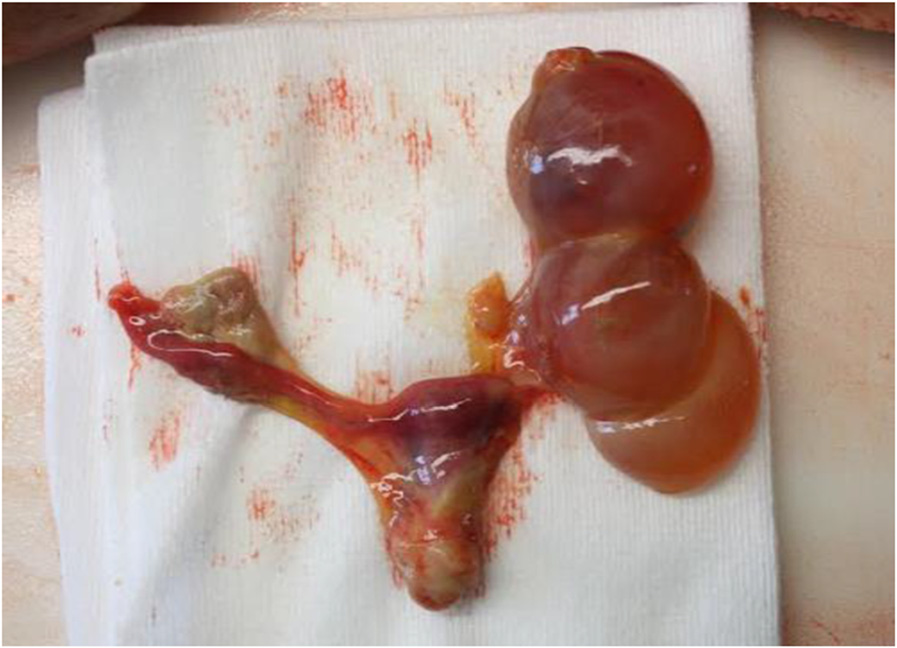
Left ovary substituted by cystic formation. Enlargement of right ovary

**Fig. 5:**
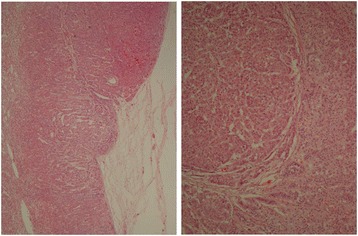
Microscopic examination confirmed micronodular hyperplasia at adrenal glands

An anatomopathological examination was performed. Macroscopic observation of her *habitus internus* (internal general physical appearance) revealed lungs with increased consistence compatible with pneumonia, heart with biventricular hypertrophy, liver with chronic congestion abnormalities and increased weight, and cortical suprarenal increased thickness. Her ovary abnormalities were most remarkable, with her right ovary 3cm long and her left ovary replaced by a cystic formation of 5.5cm greater diameter (Fig. 4). At microscopic examination, micronodular hyperplasia of the adrenal glands confirmed the etiology of Cushing’s syndrome (Fig. 5).

## Discussion

Cushing’s syndrome is the rarest of endocrine abnormalities found in MAS, and is caused by activation of Gsα in the adrenal cortex, leading to cortisol overproduction. Its prevalence in patients with fibrous dysplasia registered at the National Institutes of Health (NIH) was 7.1% [6]. Its occurrence in the neonatal period causes severe growth failure, poor muscle tone, muscular hypoplasia and hypertension; it is associated with increased and early mortality due to opportunistic infections, hence the importance of prophylactic treatment, notably for *Pneumocystis* species [7].

Brown et al. reviewed all the published cases of Cushing’s syndrome in MAS (*n* = 30) and listed the following signs and symptoms: small for gestational age (50%), round facies (66.7%), failure to thrive (60%), hypertension (33.3%), nephrocalcinosis (30%), hirsutism (26.6%), hyperglycemia (20%), and linear growth arrest (10%). Additional clinical features included liver disease, such as cholestasis or elevated transaminases (36.7%), and heart disease, such as cardiomyopathy (26.7%) [6].

In the present case, Cushing’s syndrome is likely to have manifested *in utero*, since the baby was born small for gestational age and profound hypotonia most probably as a result of prolonged exposure to hypercortisolism. The triad of failure to thrive, hypertension and hyperglycemia is the result of adrenal gland hyperplasia leading to high levels of serum cortisol. It is also possible that her comorbidities, such as hyperthyroidism and heart disease, contributed to her failure to gain weight.

Although Cushing’s syndrome can resolve spontaneously, many cases necessitate medical or surgical treatment [5, 6]. No long-term effective medical treatment for ACTH-independent Cushing’s syndrome is available. It is difficult to recognize which patients could safely be monitored and treated medically and which could benefit from adrenalectomy. We decided for medical treatment with metyrapone as our patient had cholestatic hepatitis that contraindicated the use of ketoconazole. In particularly sick children, medical treatment with metyrapone may gain time until the child is healthy enough for surgery [7]. Metyrapone was effective and well tolerated (Fig. 3). Therefore we agree with other authors that it is probably most appropriate to treat with metyrapone until a child is a suitable candidate for surgery.

Long-term sequelae of Cushing’s syndrome in MAS comprise a significantly increased prevalence of cognitive disorders, including specific learning or speech disorders such as speech apraxia, and global developmental delay [6]. These children need developmental follow-up.

A case–control analysis of patients with MAS with and without Cushing’s syndrome revealed that the presence of Cushing’s syndrome was correlated with an increased number of clinical features of MAS, such as CAL spots, precocious puberty, hyperthyroidism, cognitive disorders and total number of MAS manifestations [6]. An alternative explanation is that mutated Gsα is present in multiple organs as part of the mosaic MAS distribution, with Cushing’s syndrome signaling a greater total body mutation burden and consequently associating with more severe phenotypes.

In fact, Cushing’s syndrome usually occurs in patients with MAS with significant involvement of multiple other tissues. Table 1 represents the previously reported cases of Cushing’s syndrome in patients with MAS associated with heart and liver disease, as described in the presented case.

**Table 1:** Reported cases of McCune–Albright syndrome with Cushing’s syndrome, hepatobiliary and cardiovascular disease

Sex	Age	Cushing’s syndrome	Liver disease	Heart disease	Treatment/Outcome	Reference
F	37 days	+	Elevated LFTs	Cardiomegaly	Death (heart failure age 132 days)	[11]
M	10 months	+	Elevated LFTs, direct hyperbilirubinemia	Cardiomegaly, low ejection fraction, hypertension	Aminoglutethimide and metyrapone until age 2 years. Adrenalectomy at 2-years old. Death from cardiac arrest at age 3 years	[12]
F	2 months	+	Elevated LFTs, direct hyperbilirubinemia	Left ventricular hypertrophy	Adrenalectomy at 3 months. Death at age 2 years from anaphylaxis and/or adrenal insufficiency	[12]
M	10 weeks	+	Hepatomegaly	Cardiomegaly	NR	[13]
F	17 days	+	Elevated LFTs, direct hyperbilirubinemia, hepatomegaly	Left ventricular hypertrophy	Metyrapone. Death from pneumonia at age 4 months	Presented case

*F* female, *LFTs* liver function tests, *M* male, *NR* not reported

CAL spots are typically the first manifestation of the disease, appearing usually either at or shortly after birth [7]. As such, they can be an early clue to the diagnosis of MAS. Classically, CAL spots have irregular borders (often described as similar to the coast of Maine). To what extent the size of spots correlates to the extent of disease is controversial.

The presence of the Gsα mutation in thyroid tissue results in ligand-independent activation of the TSH/G-protein/cAMP pathway, which is known to result in both hyperplasia and hyperfunction [8]. In addition, the Gsα mutation results in increased T_4_ to T_3_ conversion, which accounts for the T_3_-dominant biochemical phenotype of patients with MAS with hyperthyroidism [9]. Although the development of thyroid cancer is rare, patients should be monitored with annual thyroid ultrasound [10]. Spontaneous resolution rate is impossible to predict, so some form of treatment must be initiated. In our patient, her hyperthyroidism responded quite well to methimazole.

Hepatobiliary dysfunction is included in the nonendocrine abnormalities associated with MAS and although it appears to be a rare manifestation, severe neonatal cholestasis and persistent elevated serum liver enzymes have been described [11–14]. The mechanism by which activated Gsα might cause hepatobiliary dysfunction remains unknown.

Hepatitis is more pronounced after birth, has laboratory manifestations consistent with cholestasis, progressively wanes with age but usually persists into adulthood (albeit mild) and is virtually never associated with a functional defect in the synthesis of important hepatic factors [14]. Both extra and intrahepatic bile ducts were patent in our patient, confirming that the defect lies at the level of the hepatocyte and/or biliary canalicular membrane, with the abnormal protein possibly interfering with the secretion of normal biliary components. The outcome of liver disease in previously described patients is not known [12].

Weinstein et al. demonstrated that cardiac Gsα mutation is more likely to be found in patients with extensive disease. Sudden death, tachycardia, ventricular hypertrophy, high-output heart failure and aortic root dilatation have all been reported in association with MAS [3]. It is not known whether it is a direct result of the presence of a primary cardiac Gsα mutation, or a secondary physiologic response to increased metabolic demand by hypercortisolism, hyperthyroidism and/or growth hormone excess. In this patient hypertension control required the association of two antihypertensives but it was only when Cushing’s syndrome was treated that the patient achieved the appropriate mean arterial pressure and myocardial hypertrophy reduced.

Respiratory distress was due to multiple factors, such as prematurity and a high cardiac output. Control of the hypercortisolism did not improve respiratory function; although this was not previously described in the literature, perhaps pulmonary involvement was also the result of Gsα mutation in the lungs.

## Conclusion

The diagnosis of MAS with Cushing’s syndrome depends on having a high index of suspicion and CAL spots may be the clue for the diagnosis. In these patients rapid assessment for significant comorbidities should be performed, including hyperthyroidism, cardiac and liver disease. Evaluation for complications of the hypercortisolism should also be conducted, including hypertension and hyperglycemia, and hypercortisolism needs to be medically managed until definitive therapy. We conclude that the poor outcome of Cushing’s syndrome in this group reflects a greater burden of mutation-carrying cells.

### Learning points

- ▶ Cushing’s syndrome in the neonatal period may suggest a diagnosis of MAS.
- ▶ CAL skin pigmentation in neonates must lead to the consideration of MAS in the differential diagnosis.
- ▶ Neonatal cholestasis is rarely associated with MAS.
- ▶ Multiple endocrine abnormalities should alert the clinician to the possibility of MAS.
- ▶ Families should be counseled that MAS is sporadic and displays no known environmental association or predilection for ethnic group.

## Consent

Written informed consent was obtained from the patient’s legal guardian(s) for publication of this case report and any accompanying images. A copy of the written consent is available for review by the Editor-in-Chief of this journal.

## Abbreviations

ACTH: Adrenocorticotropic hormone
ALT: Alanine aminotransferase
AST: Aspartate aminotransferase
CAL: Café-au-lait
cAMP: Cyclic adenosine monophosphate
γGT: Gamma-glutamyltransferase
Gsα: Alpha subunit of the stimulatory G-protein
MAS: McCune–Albright syndrome
T_3_: Triiodothyronine
T_4_: Thyroxine
TSH: Thyroid-stimulating hormone
